# Chronic high‐dose testosterone treatment: impact on rat cardiac contractile biology

**DOI:** 10.14814/phy2.14192

**Published:** 2019-07-28

**Authors:** Munthana Wadthaisong, Namthip Witayavanitkul, Tepmanas Bupha‐Intr, Jonggonnee Wattanapermpool, Pieter P. de Tombe

**Affiliations:** ^1^ Department of Physiology, Faculty of Science Mahidol University Bangkok Thailand; ^2^ Department of Cell and Molecular Physiology Loyola University Chicago Health Sciences Division Maywood Illinois; ^3^ Department of Physiology and Biophysics University of Illinois at Chicago Chicago Illinois

**Keywords:** testosterone, abuse, sarcomere, cardiomyocyte, contractility, myofilament, cardiac contractility, tension‐cost

## Abstract

Androgen therapy provides cardiovascular benefits for hypogonadism. However, myocardial hypertrophy, fibrosis, and infarction have been reported in testosterone or androgenic anabolic steroid abuse. Therefore, better understanding of the factors leading to adverse results of androgen abuse is needed. The aim of the present study was to examine the impact of high dose of androgen treatment on cardiac biology, and whether exposure duration modulates this response. Male rats were treated with 10 mg/kg testosterone, three times a week, for either 4 or 12 weeks; vehicle injections served as controls. Four weeks of testosterone treatment induced an increase in ventricular wall thickness, indicative of concentric hypertrophy, as well as increased ejection fraction; in contrast, both parameters were blunted following 12 weeks of high‐dose testosterone treatment. Cardiac myocyte contractile parameters were assessed in isolated electrically stimulated myocytes (sarcomere and intracellular calcium dynamics), and in chemically permeabilized isolated myocardium (myofilament force development and tension‐cost). High‐dose testosterone treatment for 4 weeks was associated with increased myocyte contractile parameters, while 12 weeks treatment induced significant depression of these parameters, mirroring the cardiac pump function results. In conclusion, chronic administration of high‐dose testosterone initially induces increased cardiac function. However, this initial beneficial impact is followed by significant depression of cardiac pump function, myocyte contractility, and cardiac myofilament function. Our results indicate that chronic high‐testosterone usage is of limited use and may, instead, induce significant cardiac dysfunction.

## Introduction

Androgen supplementation has been extensively discussed regarding both the advantages, and disadvantages of this therapy. While testosterone supplementation provides benefits to hypogonadic men (Anderson et al., 2016), the adverse effects of abuse of testosterone and its derivatives have also been demonstrated through numerous clinical observations. Serious adverse effects, including cardiac hypertrophy, fibrosis, and myocardial infarction have been reported for testosterone or androgenic anabolic steroid users, even leading to sudden death in some cases (Campbell et al., 1993; Montisci et al., 2012; Shamloul et al., 2014). Echocardiographic observations have shown that steroid abuse causes a reduction in both systolic and diastolic function of the heart (Baggish et al., 2010). Adverse effects have also been demonstrated in experimental animal studies. Rats treated with a high‐dose of testosterone exhibit significant myocardial hypertrophy together with an increase in apoptotic activity (Papamitsou et al., 2011). In addition, a supraphysiological dose of testosterone has been shown to inhibit nitric oxide formation through decreased expression of endothelial nitric oxide synthases (Skogastierna et al., 2014), thereby enhancing the risk of myocardial ischemia. These adverse effects of testosterone on cardiac function may significantly limit the use of this hormone.

Recently, we identified several factors that promote pathological hypertrophy in response to testosterone treatment in rats. Both the (supraphysiologic) dosage of testosterone and overall duration of administration appear to be key pathological risk factors (Pirompol et al., 2016). Our results suggested that a higher dosage and duration of testosterone administration significantly accelerates development of fibrosis. Of note, a direct impact of testosterone promoting enhanced collagen synthesis has also been observed in dermal fibroblasts (Yoo et al., 2006). Finally, testosterone induces increases in the production of angiotensin, thereby leading to enhanced neonatal cardiac fibroblast proliferation and subsequent collagen production (Yang et al., 2017). Thus, the fibrosis observed upon high‐dose testosterone supplementation may either be the direct result of testosterone itself, or a secondary compensatory effect. Apart from alterations in the extracellular matrix, supraphysiological levels of testosterone may also directly affect the cardiac contractile machinery. In normal physiology, testosterone exerts a regulatory role in maintaining cardiac myofilament function and intracellular Ca^2+^ homeostasis (Witayavanitkul et al., 2013). Lack of testosterone, on the other hand, induces decreased maximum cardiac contraction force, prolongs the cardiac relaxation time, and blunts intracellular Ca^2+^ transients. These cellular alterations are prevented by supplementation of testosterone to the physiological level. However, whether high doses of testosterone induce adverse impacts on cardiac myofilament function or intracellular Ca^2+^ activation has not yet been clearly examined.

Accordingly, in the present study we studied the impact of supraphysiological dosages of testosterone on cardiac contractile biology in a rat model. Based on our previous morphological study (Pirompol et al., 2016), we hypothesized that long‐term but not short‐term exposure to high levels of testosterone induces depressed cardiac function secondary to adverse alterations in the cardiac myocyte excitation–contraction coupling processes. Our results suggest that chronic high‐testosterone usage is of limited use and may, instead, induce severe cardiac dysfunction.

## Materials & Methods

### Animal model

This study was performed in accordance with the *Guide for the Care and Use of Laboratory Animals*, published by the National Institutes of Health using protocols approved by the Loyola University Chicago Institutional Animal Care and Use Committee. Adult male Sprague–Dawley rats weighing between 220–240 g (8 weeks of age) were obtained from Harlan Laboratories. In athletes, 1000–5000 mg of anabolic steroids per week has been reported (Pope and Katz, 1994; Parkinson and Evans, 2006; Skogastierna et al., 2014), with the caveat that the trustworthiness of the purported dose and duration of treatment is potentially doubtful. In a clinical study, administration of 600 mg testosterone per week in males has been found to increase circulating plasma levels four to six times( Bhasin et al., 1996). In animal studies, cardiovascular adverse effects were detected following 10–25 mg/kg per week treatment of testosterone for 4–10 weeks( Hassan and Kamal, 2013). Therefore, in the current study, and also based on our previous report( Pirompol et al., 2016), we employed 10 mg/kg 3 times per week for either 4 weeks or 12 weeks duration.

Following 1‐week acclimatization, rats were randomly divided into high‐dose testosterone injection or vehicle injection groups for either 4 weeks or 12 weeks of treatment duration. Testosterone propionate was injected subcutaneously three times per week (10 mg/kg body weight). Control groups were injected with ethyl oleate at a similar volume.

### Two‐Dimensional transthoracic echocardiography

Following 4 or 12 weeks of treatment, cardiac structure and function were evaluated using echocardiography (Vision Sonics Vevo2100; transducer frequency: 13–24 Hz) under isoflurane anesthesia with physiological monitoring (heart rate, respiration rate, temperature, ECG). Left ventricular morphology and systolic function were measured using M‐mode images at the mid‐papillary level from the parasternal long axis view of the left ventricle. Diastolic function was evaluated using pulsed wave Doppler‐guided mode with the apical four‐chamber view at the tip of the mitral valve leaflets to measure blood flow velocity through mitral valve during the diastolic phase. Tissue Doppler mode was used to evaluate diastolic function by measuring medial mitral annular tissue motion. All measurements and calculations are reported as average values from three consecutive cycles and performed according to the American Society of Echocardiography guidelines. Data were analyzed using Vevo2100^®^ imaging system software.

### Pressure–volume loop function measurements

Invasive left ventricular pressure–volume relationships were measured under isoflurane anesthesia and positive pressure ventilation. Catheters were inserted into the left ventricle through the right carotid artery. First, baseline pressure–volume relationships were recorded followed by a gradual inferior vena cava occlusion to derive a load‐independent measurement of the end‐systolic pressure–volume relation (ESPVR). All data were analyzed by LabScribe2 software.

### Sarcomere and intracellular calcium dynamics

Following induction of deep anesthesia, rat hearts were dissected and cannulated on a Langendorff apparatus. Retrograde perfusion was initiated through the aorta with an oxygenated Tyrode solution at pH = 7.4, 37°C, followed by digestion buffer containing Collagenase‐II and protease until digestion was complete. Hearts were cut into small pieces and then triturated. The isolated cells were separated by filtration through a 200 µm Nylon mesh. Extracellular [Ca^2+^] was progressively adjusted up to 1 mmol/L. Next, cardiac myocytes were incubated with 2 µmol/L acetoxymethyl ester of indo‐1 (Indo‐1/AM) for 5–10 min at room temperature. Myocytes were then washed with Tyrode solution (in mmol/L): 140 NaCl, 4 KCl, 1 MgCl_2_, 1 CaCl_2_, 10 glucose, 5 HEPES at pH 7.4 and studied within 4 h. An aliquot of the Indo‐1/AM incubated myocytes were transferred to an experimental chamber positioned on an inverted microscope (Nikon TMD). The chamber was perfused with the Tyrode solution (1 mmol/L Ca^2+^) and myocytes were electrically stimulated (20–30 mV; 1‐ms duration; 1 Hz). Intracellular Ca^2+^ transients and sarcomere length (SL) were simultaneously measured and analyzed using IonOptix software (IonOptix, LLC, Milton, MA).

### Chemically permeabilized (skinned) cardiomyocyte myofilament function

Skinned myocyte fragments were prepared from left ventricular samples as previously described (Ait Mou et al., 2008). Briefly, an aliquot of frozen (−80°C) left ventricle was thawed and homogenized in ice‐cold relaxing solution: 10 mmol/L EGTA, 1 mM free Mg^2+^, 5 mmol/L MgATP, 79.2 mmol/L K‐propionate, 12 mmol/L creatine phosphate, 100 mmol/L BES, pH 7.0, ionic strength 180 mmol/L, 2.5 µg/mL pepstatin A, 1 µg/mL leupeptin, 50 µmol/L PMSF, 10 U/mL creatine kinase, and 1% Triton X‐100 (Pierce) for 1 sec at 10,000 rpm using a homogenizer (Power‐Gen 700D; Fisher Scientific). The homogenate was centrifuged at 120*g* for 1 min at 4°C. Next, the pellet was filtered, and cells were washed with cold relaxing solution. Myocyte fragments were then chemically permeabilized (skinned) with relaxing solution containing 1% Triton X‐100 for 10–15 min, followed by two washing cycles with relaxing solution without Triton X‐100; skinned myocytes were used within 8 h. Next, a skinned myocyte was gently adhered to silicone glue‐coated needles attached to a force transducer (Sensonor) and motor (Aurora). SL was measured by video analysis and adjusted to 2.2 µm. Myocytes were superfused via a closely placed perfusion pipette, first with relaxing solution and next followed by activating solutions containing various calcium concentrations (pCa 10 ‐ 4.5).

### Simultaneous measurement of force and ATPase activity

Following measurement of the left ventricular pressure–volume relationship, hearts were immediately removed and perfused with Krebs Henseleit (KH) solution at pH 7.4. Unbranched ultrathin trabeculae were then carefully dissected from the right ventricle. Ventricular trabeculae were then skinned overnight at 4°C in relaxing buffer containing 1% Trtion‐X100 (as described above for skinned myocytes). The skinned trabeculae were next washed with cold relaxing solution without Triton X‐100 and used within 8 h. Isometric force and ATP consumption were measured simultaneously over a range of Ca^2+^ induced contraction forces as previously described (Rundell et al., 2004). Briefly, a skinned right ventricular trabecula was attached to a force transducer (KG4A; World Precision Instruments, Sarasota, FL) and a motor (model 308, Aurora) in relaxing solution; SL, as measured by laser diffraction, was adjusted to 2.2 µm. The muscle cross‐sectional area was calculated from the average dimensions based on an elliptical model. Variable force development was induced by activation solutions with pCa values ranging between 10.0 and 4.5 at 20°C. Muscles were discarded if the maximal force at the final contraction had decreased by >20% from the initial maximum force. Simultaneously, ATP consumption rate was measured using a coupled enzyme assay based on pyruvate kinase and lactate dehydrogenase with 2‐phosphoenolpyruvate and NADH as cosubstrate; the ATP consumption rate was determined by monitoring NADH UV light absorption at 340 nm.

### Myosin Heavy Chain (MHC) expression

Frozen ventricular samples were mixed and homogenized with extraction buffer containing protease inhibitors. Protein samples were loaded onto 6.5% polyacrylamide gels with 7.5 µg per lane using a Hoefer SE 600 apparatus for electrophoretic separation. Gel and running buffer composition was as previously described (Rundell et al., 2004), with minor modifications. SDS‐PAGE was performed using a fixed current of 45 mA for 6–7 h. Gels were stained with Coomassie blue.

### Measurement of phosphorylation level of myofilament proteins

Myofilament protein samples were prepared as previously described (Pandit et al., 2014). Briefly, 50 mg of frozen (−80°C) ventricular sample was mixed with 1 ml extraction buffer containing F‐60 (2 M KCl, 1 M imidazole, 0.1 M MgCl_2_) and phosphatase/kinase inhibitors at pH 7.0. Samples were mechanically homogenized for 150 sec. Next, the samples were centrifuged at 12,000g for 10 min at 4°C and the supernatant was discarded. The pellet was resuspended in 1 mL of F‐60 solution with 1% Triton X‐100 and homogenized a second time followed by centrifugation at 3000*g*. The pellet was collected, and the volume of extraction buffer was adjusted based on the weight of the pellet. The samples were kept at −80°C prior to analysis. Myofilament proteins were separated by SDS‐PAGE using 12.5% polyacrylamide gels. Phosphoprotein and total protein levels were determined with Pro‐Q Diamond phosphoprotein gel stain and Sypro Ruby gel stain (Molecular Probes, Invitrogen). Gels were imaged using a Typhoon Trio+ (GE Healthcare Life Sciences). Band density was analyzed using Totallab Quant version 1.0 (Amersham Pharmacia Biotech).

### Data analysis and statistics

Force‐ and ATPase‐pCa relationships were fit to a modified Hill equation, while tension‐cost was determined by linear fit to the force‐ATPase data (Rundell et al., 2004). Data were analyzed using independent Student’s *t*‐test after 2 x 2 Factorial ANOVA followed by Newman–Keuls multiple comparisons test; statistical significance set at *P* < 0.05. Data are presented as mean ± SE.

## Results

### General characteristics

As in our previous study, a greater seminal vesicular weight was utilized to confirm over‐activation due to high‐dose testosterone (Table 1). Interestingly, the high‐dose of testosterone used in this study induced a significant reduction of body weight after 4 weeks, but the body weight returned to normal following 12 weeks of treatment. Although heart weight and left ventricular weight significantly increased after 12 weeks of high‐dose testosterone treatment, the ratio to body weight (cardiac hypertrophic index) revealed a similar degree of hypertrophy between 4‐week and 12‐week high‐dose testosterone treatment. There was no sign of congestive heart failure following high‐dose testosterone treatment as indicated by the absence of lung edema (lung weight wet/dry ratio).

**Table 1:** General characteristics of vehicle‐ and high testosterone‐treated rats for 4 and 12 weeks.

Parameter	4 weeks		12 weeks	
	Vehicle (*n* = 11)	Testosterone (*n* = 11)	Vehicle (*n* = 11)	Testosterone (*n* = 11)
BW (g)	334 ± 9	311 ± 6	378 ± 10†	358 ± 10†
HW (g)	1.04 ± 0.04	1.16 ± 0.03*	1.08 ± 0.03	1.25 ± 0.03*
LV (g)	0.77 ± 0.03	0.79 ± 0.01	0.82 ± 0.03	0.91 ± 0.02* ^,^ †
SV (g)	0.58 ± 0.04	0.93 ± 0.03*	0.64 ± 0.05	1.11 ± 0.05* ^,^ †
Soleus weight (g)	0.120 ± 0.004	0.111 ± 0.003	0.139 ± 0.007†	0.133 ± 0.002†
HW/BW (mg/g)	3.08 ± 0.10	3.77 ± 0.08*	2.89 ± 0.04	3.50 ± 0.07* ^,^ †
LVW/BW (mg/g)	2.31 ± 0.08	2.63 ± 0.04*	2.16 ± 0.04	2.55 ± 0.06*
Lung weight wet/dry ratio	4.67 ± 0.21	4.96 ± 0.16	4.77 ± 0.18	5.17 ± 0.09

Data are shown as mean ± SE. BW, body weight; HW, heart weight; LV, left ventricular weight; SV, seminal vesicular weight.

* Indicates significant difference (*P* < 0.05) between the vehicle and treatment groups for each duration (4 or 12 weeks) or

^†^ Between groups at either 4‐ or 12 weeks of treatment, respectively, using independent t‐test after 2x2 Factorial ANOVA followed by Newman‐Keuls multiple comparisons.

### Cardiac hypertrophy and altered cardiac pump function

Echocardiographic measurements also confirmed hypertrophy of the heart in the high‐dose testosterone‐treated groups. Increases in wall thickness of the interventricular septum and left ventricular posterior wall were noted after 4 weeks of treatment (Table 2) but diminished following 12 weeks of treatment. Moreover, high‐dose testosterone treatment did not affect left ventricular chamber size, apart from changes due to aging. An increase in wall thickness following high‐dose testosterone treatment suggests cardiac remodeling toward more concentricity in the early stages of testosterone treatment, reverting toward control levels in the 12‐week‐treated rats. Of note, increases in ejection fraction and fractional shortening were observed in the hearts of 4‐week high‐dose testosterone‐treated rats, indicating overall enhanced cardiac contractility. In contrast, this positive impact on cardiac contractility was no longer observed following 12 weeks of testosterone treatment. The changes in cardiac contractility were confirmed by invasive pressure–volume loop measurements (Table 3). Of note, age *per se* did not affect cardiac contractility, and there were no changes observed in passive cardiac stiffness properties (Table 3).

**Table 2:** Echocardiographic data of vehicle‐ and high testosterone‐treated rats for 4 and 12 weeks.

Parameter	4 weeks		12 weeks	
	Vehicle (*n* = 11)	Testosterone (*n* = 11)	Vehicle (*n* = 8)	Testosterone (*n* = 11)
IVSd (mm)	1.59 ± 0.04	1.82 ± 0.04*	1.53 ± 0.05	1.70 ± 0.06
LVPWd (mm)	1.60 ± 0.03	1.85 ± 0.05*	1.55 ± 0.06	1.70 ± 0.05†
LVIDd (mm)	7.59 ± 0.13	7.27 ± 0.13	8.35 ± 0.17†	8.20 ± 0.14†
LVIDs (mm)	4.97 ± 0.13	4.33 ± 0.14*	5.69 ± 0.17†	5.54 ± 0.17†
LV mass (mg)	706 ± 27	782 ± 25	769 ± 41	863 ± 25^*,†^
Relative wall thickness	0.42 ± 0.01	0.51 ± 0.02*	0.37 ± 0.01	0.42 ± 0.02†
Ejection fraction (%)	60.4 ± 1.4	67.6 ± 1.0*	60.7 ± 1.5	57.4 ± 1.3†
Fractional shortening (%)	34.2 ± 1.5	40.1 ± 1.3*	32.7 ± 0.9	32.6 ± 1.1†
Stroke volume (µL)	278 ± 17	294 ± 12	336 ± 8^†^	296 ± 11*
Heart rate (bpm)	329 ± 10	465 ± 87	386 ± 57	316 ± 9
Cardiac output (ml/min)	91 ± 6	196 ± 24	130 ± 20	95 ± 3
MV E/A	1.35 ± 0.05	1.58 ± 0.09*	1.23 ± 0.07	1.45 ± 0.08
E/e’	14.9 ± 1.7	18.7 ± 3.7	14.8 ± 1.0	20.2 ± 1.7

Data are shown as mean ± SE. IVSd, interventricular septum thickness at diastole; LVPWd, left ventricular posterior wall thickness at diastole; LVIDd, left ventricular inner diameter at diastole; MV E/A, ratio of mitral peak velocity of early filling (E) to mitral peak velocity of late filling (A); E/e’, the ratio between early mitral inflow velocity and mitral annular early diastolic velocity.

*,† Indicate the level of significance as in Table 1.

**Table 3:** Pressure–volume Loop function data of vehicle‐ and high testosterone‐treated rats for 4 and 12 weeks

Parameter	4 weeks		12 weeks	
	Vehicle (*n* = 10)	Testosterone (*n* = 11)	Vehicle (*n* = 10)	Testosterone (*n* = 11)
ESP, mmHg	114 ± 4	116 ± 3	117 ± 4	114 ± 3
EDP, mmHg	11.3 ± 0.95	9.5±0.83	9.3 ± 1.06	8.7±0.68
dP/dt Max, mmHg/s	6155 ± 193	6472 ± 353	6470 ± 265	5992 ± 138
dP/dt Min, mmHg/s	6361 ± 376	6257 ± 353	6210 ± 340	5814 ± 178
Tau, msec	10.2 ± 0.4	11.0 ± 0.4	11.2 ± 0.5	11.2 ± 0.3
EDV, µL	296 ± 26	348 ± 26	381 ± 32	326 ± 45
SV, µL	218 ± 16	258 ± 21	282 ± 31	229 ± 29
Heart rate (bpm)	319 ± 6	311 ± 8	305 ± 10	315 ± 10
Cardiac output (ml/min)	71 ± 5	81 ± 7	84 ± 8	73 ± 10
ESPVR slope, mmHg/µL	0.44 ± 0.04	0.75 ± 0.12*	0.38 ± 0.05	0.47 ± 0.07†
Passive stiffness, µL^‐1^	0.003 ± 0.001	0.003 ± 0.001	0.004 ± 0.001	0.005 ± 0.001

Data are shown as mean ± SE. ESP, end‐systolic pressure; EDP, end‐diastolic pressure; dP/dt Max, the maximum rate of pressure development; dP/dt Min, the maximum rate of pressure decay; Tau, relaxation time constant; EDV, end diastolic volume; SV, stroke volume; ESPVR, end‐systolic pressure–volume relationship; passive stiffness, slope of the diastolic pressure–volume relationship.

*,† Indicate the level of significance as in Table 1.

### Sarcomere and intracellular calcium dynamics; myocyte force development

**Figure 1:**
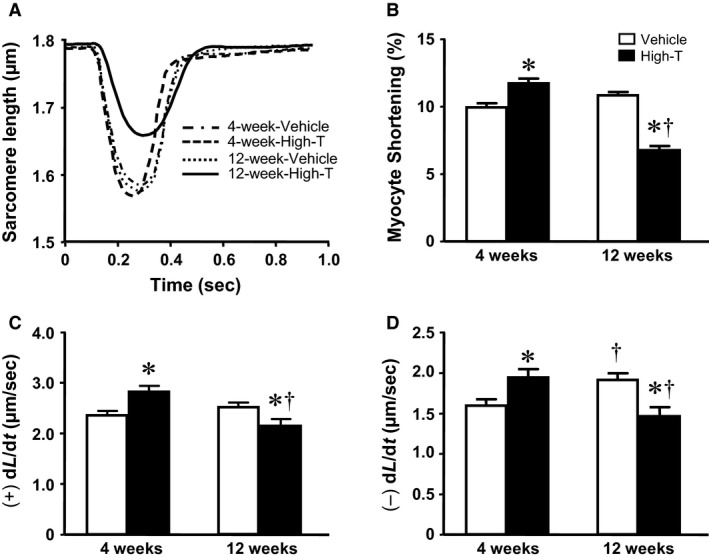
Contraction and relaxation in isolated intact cardiomyocytes. (A) Traces represent sarcomere length (SL) shortening and relengthening of cardiomyocytes from vehicle and 10 mg/kg testosterone‐treatment in rats for 4 and 12 weeks. Bar graphs demonstrate (B) normalized SL shortening; (C) rate of SL shortening, (+)dL/dt; and (D) rate of SL relengthening, (‐)dL/dt. Data are mean ± SE from ~ 50 cells (from five hearts) in each group. *, ^†^ indicates significantly different (*P* < 0.05) between the vehicle and treatment groups for each duration (4 or 12 weeks) or, † between groups at either 4 or 12 weeks of treatment, respectively, using independent t‐test after 2 x 2 Factorial ANOVA followed by Newman–Keuls multiple comparisons.

**Figure 2:**
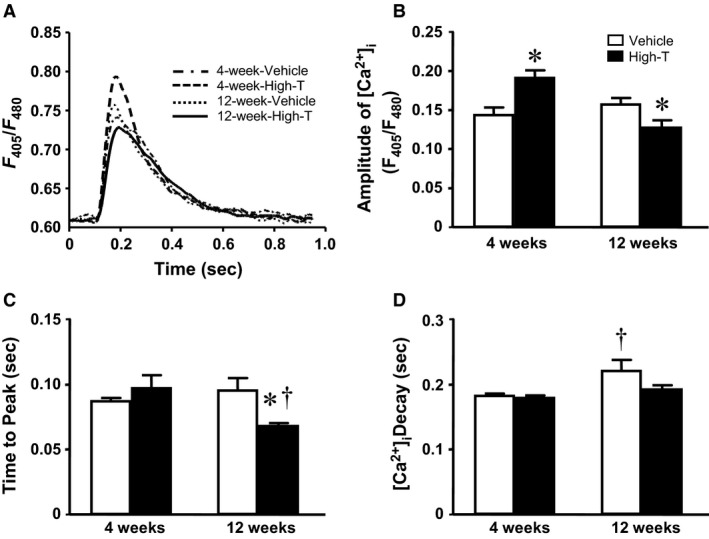
Intracellular Ca^2+^ transients in isolated cardiomyocytes. (A) Traces represent intracellular Ca^2+^ transients of electrically stimulated cardiomyocytes in testosterone‐treated rats and vehicle control. Bar graphs summarize: (B) amplitude of intracellular Ca^2+^ transients; (C) time to peak Ca^2+^; and (D) time of Ca^2+^ decay. Data are mean ± SE from ~ 50 cells (from five hearts) in each group. *, ^†^ as in Figure 1.

**Figure 3:**
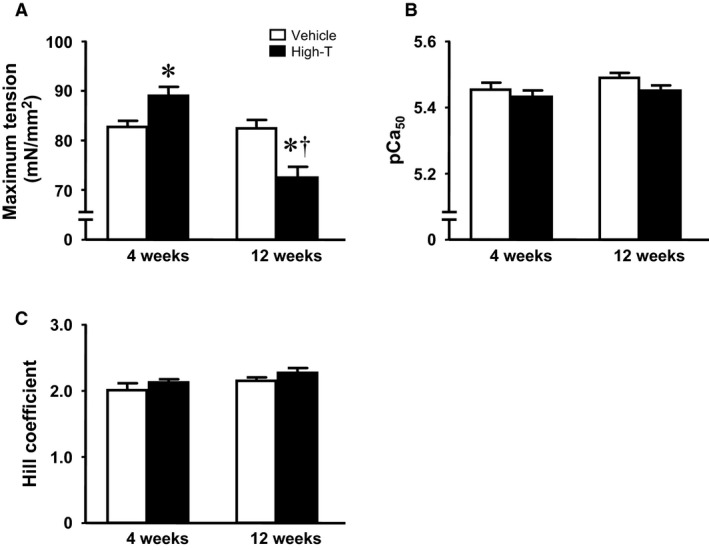
Myofilament calcium responsiveness measured in chemically permeabilized isolated myocytes from testosterone‐treated rats and vehicle control. (A) Maximum, Ca^2+^ saturated tension development. (B) Calcium sensitivity, indexed by –log[Ca^2+^] at half maximum tension. (C) Cooperativity indexed by the Hill coefficient. Data are mean ± SE from 17–18 cells (from five hearts) in each group. *, ^†^ as in Figure 1.

To asses cellular mechanisms underlying the observed changes in cardiac contractility induced by high‐dose testosterone treatment, mechanical properties of cardiomyocytes and myocardial tissues were examined. Membrane intact electrically stimulated cardiomyocytes from the 4‐week testosterone‐treated group exhibited a significant increase in SL shortening compared to the 4‐week vehicle control group (11.2 ± 0.4% vs. 10.0 ± 0.2%, Fig. 1A and B). While age *per se* did not affect SL shortening, this parameter significantly decreased in myocytes isolated from 12‐week testosterone‐treated rats (6.2 ± 0.3%). Likewise, SL shortening velocity was increased following 4 weeks of high‐dose testosterone treatment and was significantly depressed in the 12‐week high‐dose testosterone‐treated group (Fig. 1C), as was the rate of sarcomere relengthening (Fig. 1D). The Ca^2+^ transient amplitude was increased following 4 weeks of treatment (F_405/480_: 0.17 ± 0.01 versus 0.13 ± 0.01 in vehicle control but decreased following 12 weeks of treatment (Fig. 2 A and B). The time to peak intracellular Ca^2+^, a parameter associated with SL shortening velocity, was not changed in the 4‐week treatment group but decreased following 12‐week of high‐dose testosterone treatment (Fig. 2C). There was no difference in the rate of Ca^2+^ transient decay in the week 4 treatment groups, whereas this parameter was significantly increased in the 12‐week vehicle‐treated group, but not in high‐dose testosterone treatment group. Another factor that can affect cardiac contraction is myofilament function (force generating capacity and Ca^2+^ sensitivity). These parameters were determined using skinned cardiomyocytes (Fig. 3). Cardiac cells from 4‐week high‐dose testosterone‐treated rats displayed an increase in maximum force development compared to the vehicle control group, while this parameter significantly depressed following 12 weeks of high‐dose testosterone treatment (Fig. 3A). Of note, in a previous study (Pirompol et al., 2016) we did not observe increased maximum myofilament force development following short‐term high‐dose testosterone treatment. The reasons underlying our current contrasting results at the 4‐week time point are not entirely clear, but may be related to differences in muscle preparation or skinned muscle solution compositions. In contrast, neither myofilament Ca^2+^ sensitivity (Fig. 3B) nor cooperativity of force development (Fig. 3C; Hill Coefficient) were affected by high‐dose testosterone treatment. Note that the Hill coefficient was measured without strict sarcomere length control during the contraction (Dobesh et al., 2002), which may cause underestimation of this parameter.

### ATPase activity and tension‐cost

**Figure 4:**
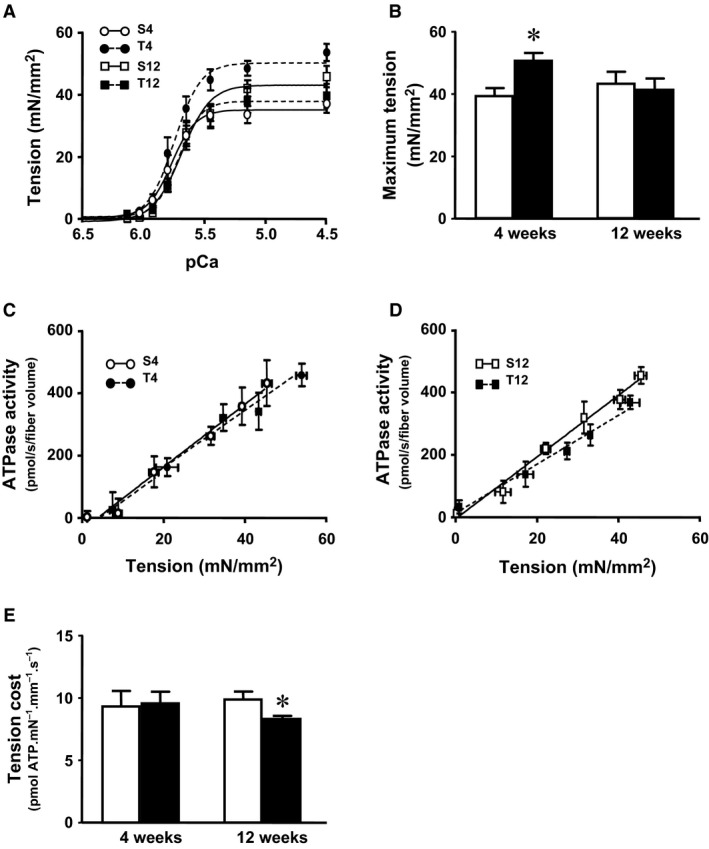
ATPase activity, and tension‐cost measured in chemically permeabilized isolated right ventricular trabeculae from testosterone‐treated rats and vehicle control. (A) Force‐pCa relationships and (B) Average maximum tension. (C) & (D) Tension‐ATPase activity relationship in 4‐week and 12‐week treated groups, respectively. (E) Average tension‐cost (slope of tension‐ATPase activity relationship). Data are mean ± SE from 7–8 trabeculae (from four hearts) in each group. *, ^†^ as in Figure 1.

Cross‐bridge kinetics parameters were assessed in skinned multicellular trabeculae isolated from the right ventricle (Figure 4). The ATP consumption rate was measured as a function of myofilament force production (tension‐cost) in skinned trabecular preparations over a range of activating [Ca^2+^]. In general, maximum force development, myofilament Ca^2+^ sensitivity and cooperativity (Fig. 4A and B) mirrored results obtained in skinned myocytes (cf. Fig. 3), although depressed force development in the 12‐week group did not reach statistical significance. Tension‐cost, the relationship between ATPase activity and force development was not affected in the 4‐week treatment group (Fig. 4C), but significantly reduced in the 12‐week high‐dose testosterone‐treated group (Fig. 4D), as summarized in Figure 4E. Suppressed tension‐cost implies that there is a reduction in the detachment rate of actively cycling cross bridges following chronic high‐dose testosterone treatment. Note that maximum force development estimates differed between multicellular (Fig. 4) and single myocyte skinned myocardium (Fig. 3.) preparations, as has been observed previously by both others and us. The underlying causes for this phenomenon are not entirely clear. It may be related to the absence of extracellular matrix and the relative uncertainty in determining cross‐sectional area in the isolated single cardiac myocyte preparation.

### Myosin isoform expression and contractile protein phosphorylation

**Figure 5:**
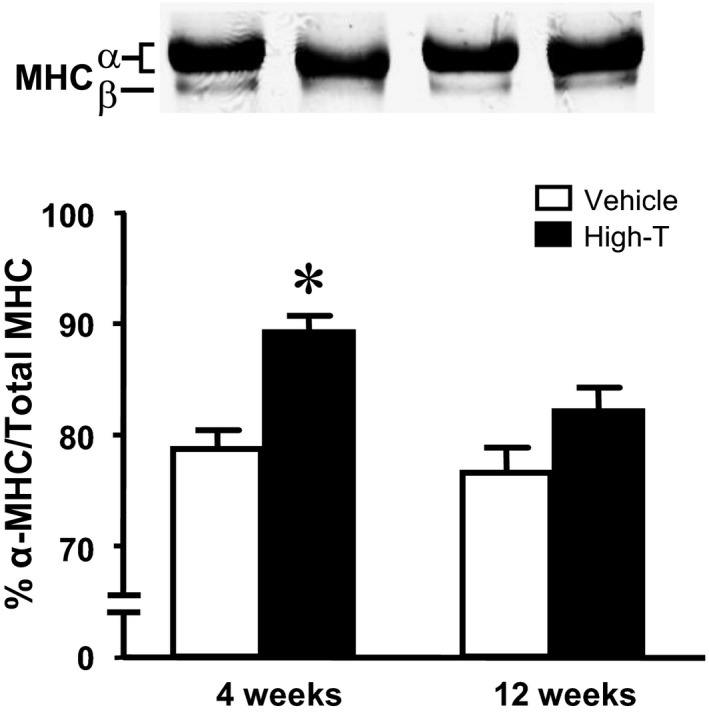
Impact of high‐dose testosterone treatment on the expression of cardiac myosin heavy chain (MHC) isoforms. Samples were analyzed by SDS‐PAGE as described in Methods. The top panel shows representative MHC protein bands of left ventricular samples from vehicle and 10 mg/kg testosterone‐treated rats for 4 and 12 weeks. Bar graphs summarize normalized α‐MHC per total MHC protein. Data are mean ± SE from 10–11 hearts each group. *, ^†^ as in Figure 1.

**Figure 6:**
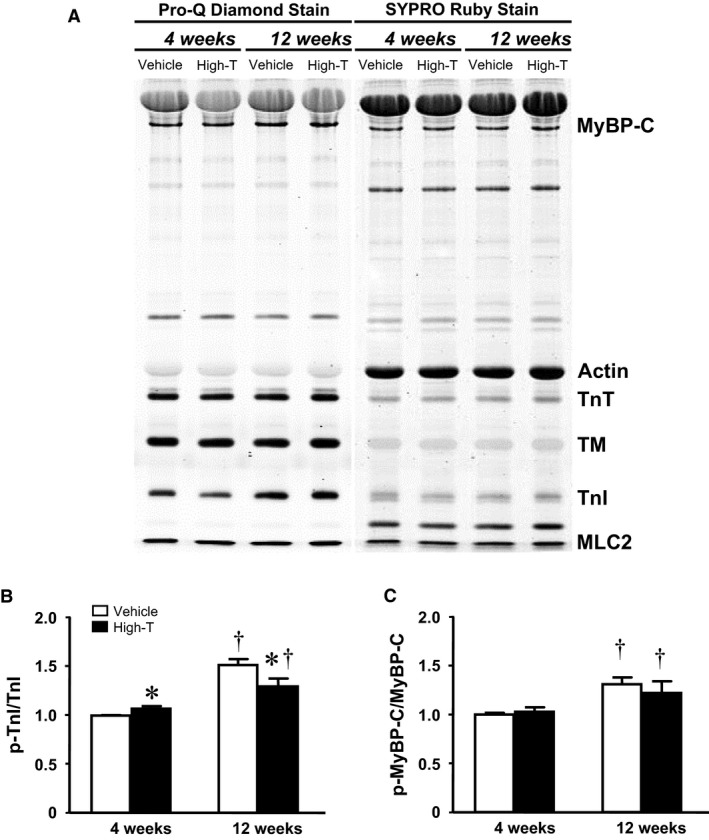
Cardiac myofilament protein phosphorylation. Left ventricular samples from vehicle control and 10 mg/kg testosterone‐treated rats for 4 and 12 weeks were analyzed by SDS‐PAGE. (A) Representative phosphorylated and total protein bands as stained with Pro‐Q diamond (left) and SYPRO ruby (right), respectively. Bar graph summarizing relative phosphorylation level of (B) cardiac troponin I (TnI) and (C) myosin binding protein C (MyoBP‐C). Data are mean ± SE from 7–8 hearts each group. The phosphorylation signal from TnI the 4‐week vehicle control group was used for normalization in each gel. *, ^†^ as in Figure 1

To asses possible molecular causes for the observed changes cardiac contractile biology parameters, myofilament myosin isoform composition and phosphorylation of cardiac thick and thin filament proteins were examined. SDS‐PAGE analysis revealed an increase in the ratio of α‐MHC to total MHC following 4‐week high‐dose testosterone treatment as compared to vehicle control (Fig. 5). The increase in the MHC ratio returned to levels seen in the control group in hearts from 12‐week high‐dose testosterone‐treated rats. There were no any other changes in myofilament protein composition following high‐dose testosterone treatment (Fig. 6A; Sypro). Using ProQ‐diamond fluorescent staining of phosphoproteins, a significant increase in phosphorylation of troponin I (TnI) was detected in samples from 4‐week high‐dose testosterone‐treated rats (Fig. 6B). Unexpectedly, phosphorylation of TnI also increased with age, but the level in preparations from 12‐week high‐dose testosterone‐treated rats was significantly lower compared to the 12‐week vehicle control group. Moreover, an increase in phosphorylation of myosin binding protein C (MyBP‐C) was observed in the hearts of both 12‐week vehicle and high‐dose testosterone‐treated rats, indicative of a purely age dependent phenomenon (Fig. 6C).

## Discussion

Our study provides new information regarding the cardiac pathophysiological impact of high‐dose testosterone treatment. Specifically, we studied cardiac hemodynamics using noninvasive echo cardiography and invasive pressure–volume loop analysis, isolated single myocyte sarcomere dynamics and intracellular calcium transient, isolated skinned myocyte myofilament function, and tension‐cost measurements in skinned multicellular isolated myocardium. Our results indicate that prolonged high‐dose testosterone treatment induces cardiac dysfunction, possibly because of the induction of hypertrophic pathways. Short‐term treatment with high levels of testosterone induced concentric hypertrophy, and an unexpected increase in contractile function. The increased inotropy appeared to be caused by an increased intracellular Ca^2+^ transient, as well increased myofilament force producing capacity. However, this positive inotropy reversed following long‐term high‐dose testosterone treatment, concomitant with depressed myofilament function that may be the result of reduced cardiac troponin‐I phosphorylation. Our findings suggest that the development of pathological cardiac hypertrophy following long‐term high‐dose testosterone treatment is not the direct consequence of genomic activation by testosterone, but rather mediated through sequential maladaptation.

Due to the presence of androgen receptors on the cardiac myocytes (Marsh et al., 1998; Dart et al., 2013), a genomic effect of testosterone on myocardial function has been postulated. It is well accepted that the physiological level of testosterone plays a beneficial role in cardiac contractile biology (Curl et al., 2009; Witayavanitkul et al., 2013). Besides the hypertrophic effect, Golden and coworkers demonstrated that acute high‐dose testosterone treatment in cultured rat cardiac myocytes induced increases in amplitude and velocity of cell shortening (Golden et al., 2005). This positive inotropy indicates a nongenomic, direct impact of a high‐dose application of testosterone. The increase in the phosphorylation level of cardiac TnI observed in the present study following 4‐week high‐dose testosterone treatment also suggests a direct, nongenomic, posttranslational signal transduction modulation induced by the hormone (cf. Fig. 6). Numerous studies have documented the adverse effects of high‐dose testosterone and anabolic steroids following long‐term administration (D'Andrea et al., 2007; Baggish et al., 2010). That is, increased left ventricular mass and smaller left ventricular chambers were apparent in athletes using steroids, indicating a disproportionate cardiac hypertrophy (Dickerman et al., 1997). Interestingly, in our analysis a concentric hypertrophy that was detected at 4 weeks of high dose testosterone treatment reverted following 12 weeks high‐dose treatment (cf. Table 1). We speculate that cardiac remodeling reverts to maladaptation following extended, chronic exposure to testosterone. This could explain the decreased cardiac contractility we observed following the prolonged treatment with high‐dose testosterone (cf. Tables 2 and 3). Our previous report demonstrated the presence of fibrosis following 12 weeks of high‐dose testosterone treatment, suggesting the possibility of myocardial infarction (Pirompol et al., 2016). A diminished cardiac capillary density following testosterone (3 mg/kg body weight) or androgenic anabolic steroid administration during exercise training has indeed been observed (Tagarakis et al., 2000, 2000). It has also been reported that chronic use of androgenic anabolic steroid is associated with a high incidence of coronary calcification (Santora et al., 2006). Thus, the increased cellular calcium signaling, and concomitant increased cardiac contractility induced by short term (4‐week) treatment with high‐dose testosterone may cause the development of cardiac pathology we see at the 12‐week time point. On the other hand, insufficient cardiac perfusion may underlie the transition to reduced cardiac structure and function following long‐term chronic high‐dose testosterone treatment.

In the current study, short‐term 4‐week high‐dose treatment with testosterone was associated with an increase in cardiac contractility *in‐situ* as well as enhanced contractility at the cellular level (cf. Fig. 1). The physiological impact of testosterone in altering cellular Ca^2+^ homeostasis may be due to alterations in the expression of SERCA, NCX, or calsequestrin 2 (Curl et al., 2009; Sebag et al., 2011; Witayavanitkul et al., 2013). Moreover, acute application of physiological doses of testosterone has been shown to increase the intracellular Ca^2+^ transient amplitude in the HL‐1 cardiac cell line (Hsu et al., 2015). Likewise, treatment with testosterone, at 100 nmol/L for 24 h, significantly enhances whole‐cell *I*_(Ca, L)_ of isolated rat cardiomyocytes due, in part, to increased expression of the alpha 1C subunit of L‐type calcium channel leading to enhanced single‐channel activity (Er et al., 2007). Increased cytosolic Ca^2+^ following high‐dose testosterone application in isolated cardiomyocytes has been proposed as a potent hypertrophic stimulus (Vicencio et al., 2006). Moreover, high‐dose testosterone can promote proapoptotic activity in many cell types (Lopes et al., 2014; Nascimento et al., 2015). Therefore, it is likely that a combination of such mechanisms ultimately leads to the development of cardiac pathology with pathways converging into depressed cellular Ca^2+^ homeostasis (cf. Fig. 2) and reduced myofilament function (cf. Figs. 3 and 4).

Myofilament force development capacity and cross‐bridge cycle kinetics (tension‐cost) were significantly reduced following 12 weeks high‐dose testosterone treatment, which likely contributed to the reduced cardiac and cellular contractility seen at this time point. Changes in myofilament function can be induced both by pre‐ and posttranslational modifications to myofilament proteins, such as isoform expression and phosphorylation state. Testosterone has been shown to indirectly regulate MHC expression causing a shift of MHC isoforms toward a greater abundance of α‐MHC (Morano et al., 1990; Thum and Borlak, 2002). The altered MHC ratio correlated with increased maximum myofilament force development in the short‐term (4 weeks) treatment group, but whether this causally contributed to enhanced force is not clear at this point. It should be noted that large mammals, in contrast to rodents, predominantly express β‐myosin (Rundell et al., 2005). Hence, translation of the current myosin isoform results obtained in rodents to the human should be made with caution. The reversal of the MHC ratio following long‐term treatment with high‐dose testosterone indicates the possibility of androgen receptor desensitization. Recent observations have demonstrated that anabolic androgenic steroids abusers exhibited a significant suppression in gonadotropin level (Rasmussen et al., 2017), which may downregulate androgen receptor expression (Su et al., 2013). The decrease in cross‐bridge kinetics could be another representation of the development of cardiac pathology. Although a lower tension‐cost indicates more efficient ATP utilization (Rundell et al., 2004; Rundell et al., 2005), decreased tension‐cost has also been found in many pathological models (Rundell et al., 2004; Zobel et al., 2007).

It has been hypothesized that the reduction in tension‐cost is an adaptive response to maintain contractility and reduce cardiac energy demand, which may relate to altered MHC isoform expression (cf. Fig. 5) or altered contractile protein phosphorylation (cf. Fig. 6) following the long‐term high‐dose testosterone treatment. Of note, it has been shown that contractile protein phosphorylation is a potent modulator of cross‐bridge cycling kinetics (Pyle et al., 2002; Scruggs et al., 2009; Sumandea et al., 2009). The specific contractile protein phosphorylation targets sites responsible for the depression of tension‐cost, however, cannot be determined from our current study.

In conclusion, our present results demonstrate that short‐term administration of testosterone induces a positive impact on cardiac contractile function, even at supraphysiological concentrations. However, the duration of treatment becomes a key factor in determining the outcome. Prolonged high‐dose treatment with the hormone led to maladaptation and depressed cardiac pump function and cellular contractility mediated, in part, by depressed cellular calcium homeostasis and myofilament function. Further study is required to fully understand the cellular and molecular mechanisms underlying the cardiac pathology associated with supraphysiological exposure to testosterone. Regardless, our current findings support the notion of a severe negative cardiac impact of nontherapeutic use of androgenic steroids.

## Conflict of Interest

There are no conflicts of interest to report for this study.
